# Supplementary material for: The evolutionary impact of childhood cancer on the human gene pool
Source: Nat Commun. 2024 Feb 29;15:1881. doi: 10.1038/s41467-024-45975-9 (PMC10904397; doi:10.1038/s41467-024-45975-9)
Supplement: Supplementary file 1 — Supplementary Information [file 41467_2024_45975_MOESM1_ESM.pdf]

SUPPLEMENTARY MATERIALS: The Evolutionary Impact of Childhood Cancer on the Human Gene Pool

Supplementary Table 1: Categorized level of evidence of constraint for loss-of-function variants. Blod font denotes a cancer predispostion phenotype driven by gain-of-function gene variation. AD, autosomal dominant, XLR, X-linked recessive, pCPS, pediatric cancer predisposition syndrome, AR, autosomal recessive.

**AD/XLR pCPS genes [n = 62]**			
Constrained	n = 38	61% of total	*APC, BAP1, CDC73, CREBBP, CTR9, CTLA4, DICER1, ETV6, EZH2, FBXW7, GATA2, GPC3, IKZF1, MEN1, NF1, NF2, NSD1, PAX5, PHOX2B,* ***PIK3CA****, RB1, REST,* ***RET****, SETBP1, SMARCA4, SMARCB1, SMARCE1, STK11, TRIM28, TSC1, TSC2, WAS, WT1, PTCH1, SUFU, TRIP13,* ***BRAF****,* ***PTPN11***
Likely constrained	n = 15	25% of total	*AIP,* ***ALK****, CDH1, CDKN1C, CDKN2A, CEBPA, FAS, PTEN, RUNX1, SH2D1A, TP53, CBL,* ***RIT1****, FH, SDHD*
Likely not constrained	n = 4	6% of total	*GPR161,* ***HRAS****, SDHB, VHL*
Not constrained	n = 5	8% of total	*IKBKAP,* ***SAMD9****,* ***SAMD9L****, SDHA, SDHC*
**AR pCPS genes [n = 23, data missing for one]**			
Constrained	n = 2	9% of total	*DIS3L2, MSH2*
Likely constrained	n = 1	4% of total	*MSH6*
Likely not constrained	n = 8	35% of total	*CD27, HAVCR2, ITK, LIG4, TRIM37, USB1, MLH1, BUB1B*
Not constrained	n = 11	48% of total	*ABCB11, ATM, BLM, CD70, NBN, RECQL4, SBDS, BRCA2, FANCA, PALB2, PMS2*

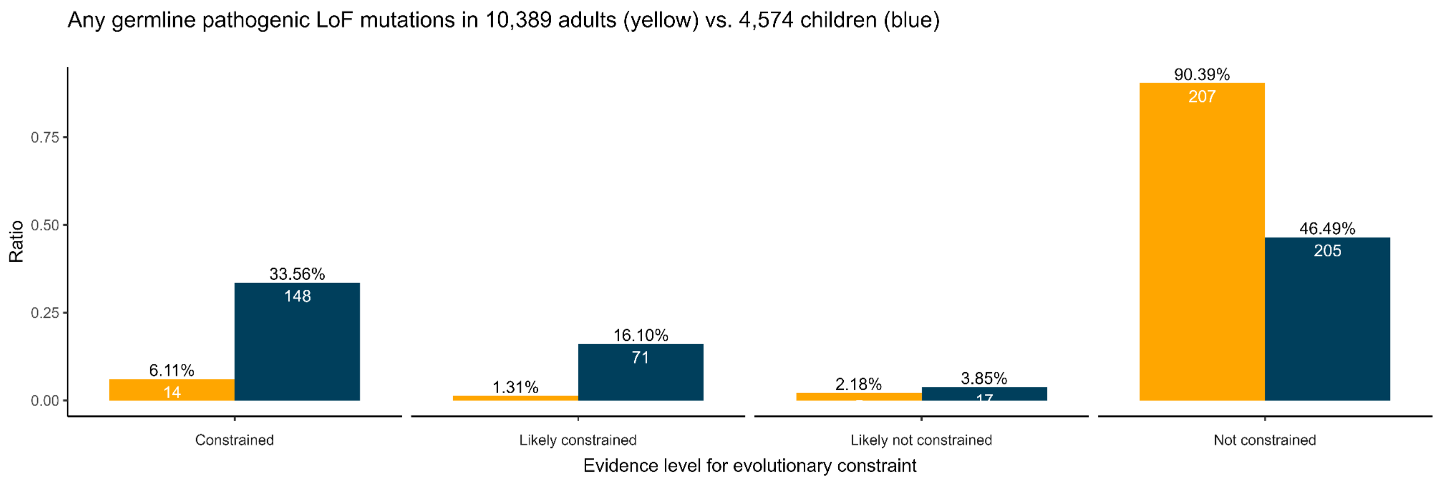

Supplemental Figure 1: Bar plot showing the level of constraint for pathogenic loss-of-function (LoF) variants reported as pathogenic in adults (in yellow) and in children (in blue) in pan-cancer cohorts. Ratio on the y-axis refers the the proportion of all observed variants reported as pathogenic in each group. Source data are provided as a Source Data file.

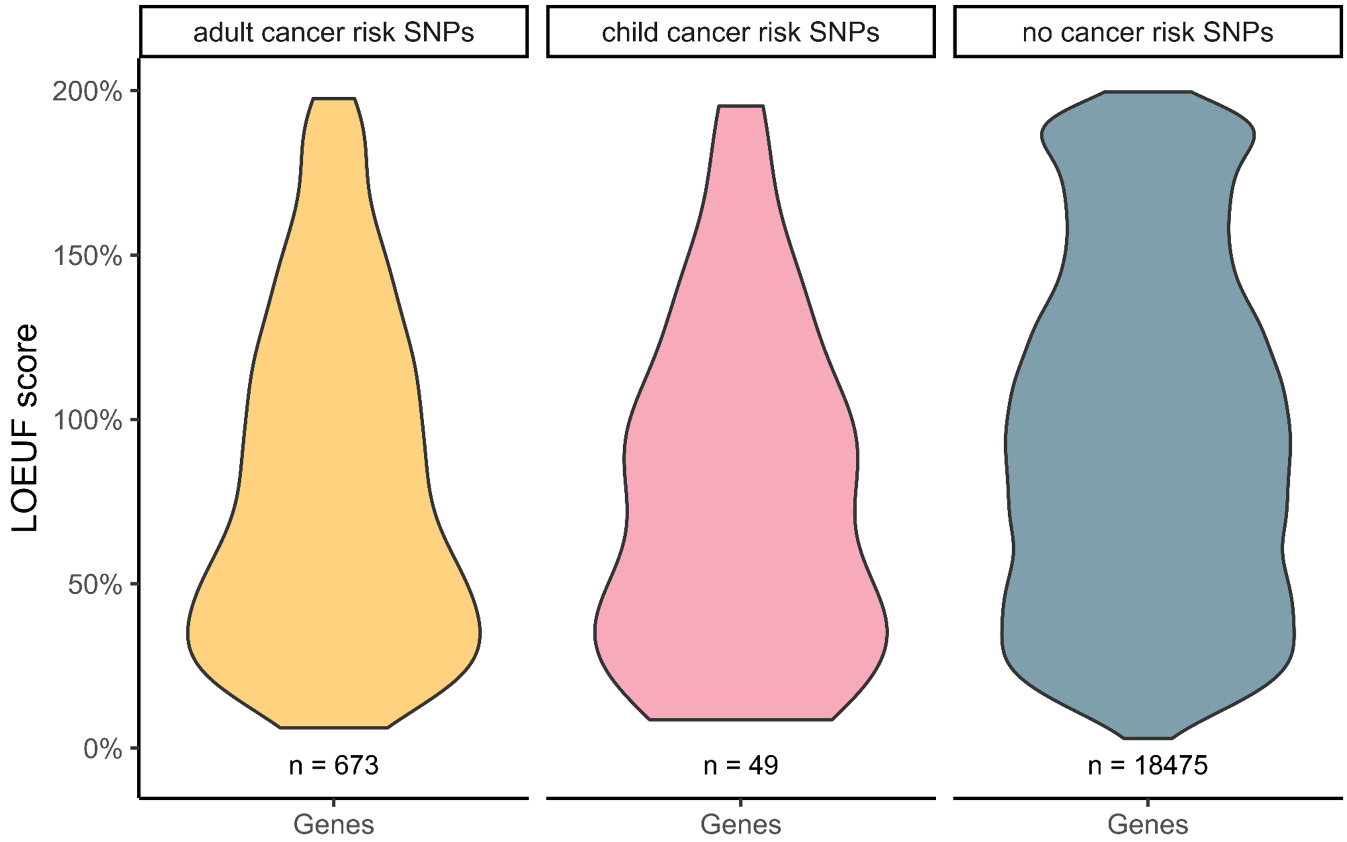

Supplementary Figure 2: The plot represents the distribution of LOEUF scores for sets of genes based on the presence of single nucleotide polymorphisms (SNPs) associated with cancer risk. Violin plots are used to show the distribution of scores for each gene set, with the width of the plot corresponding to the density of data points at each value. Colors indicate gene set. Embedded labels indicate the number of genes (n) for each gene set. Source data are provided as a Source Data file.

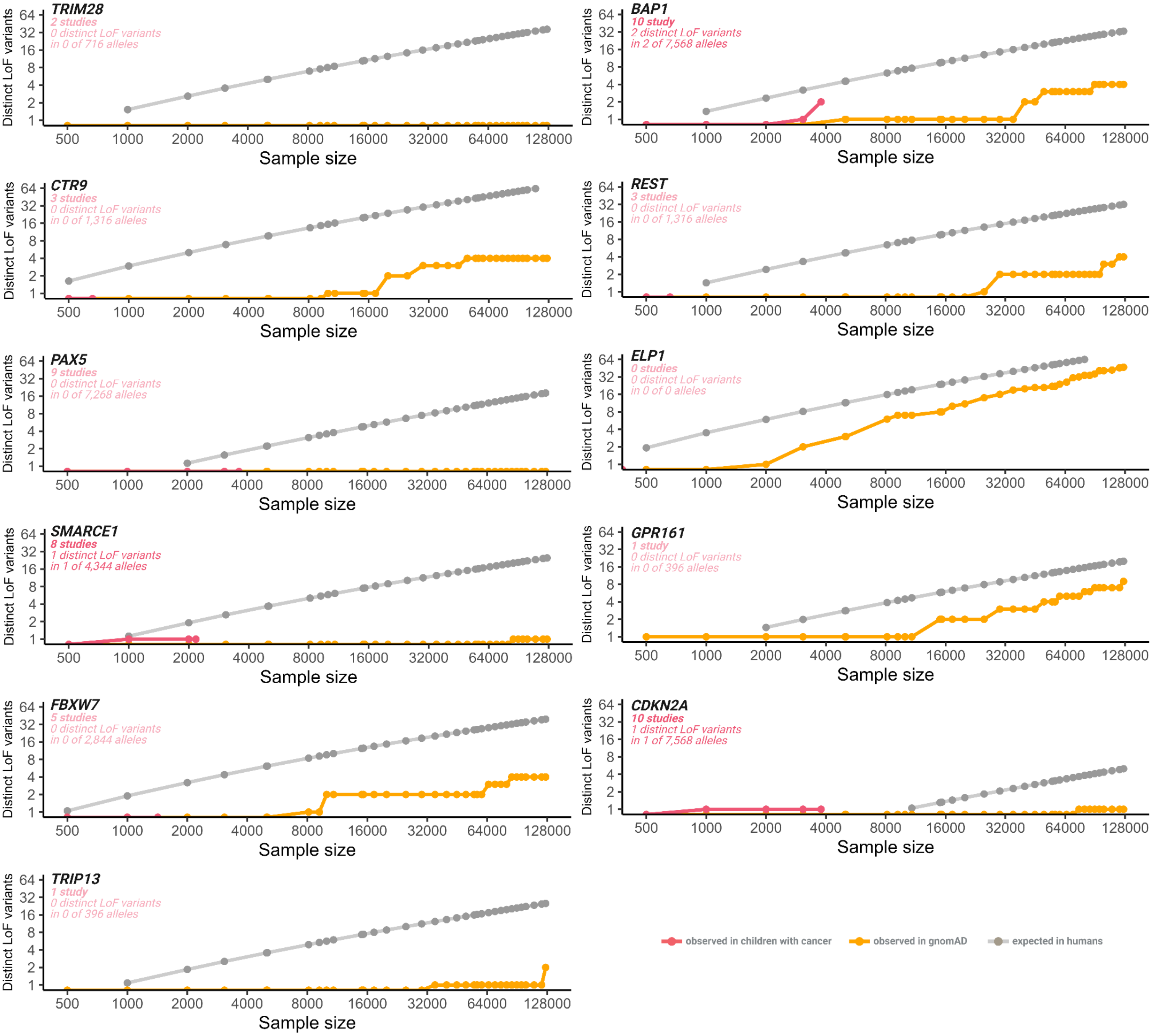

Supplementary Figure 3: Log-scaled point graphs showing number of distinct LoF variants at various sample sizes, with colors representing gnomAD data, pediatric pan-cancer data or expected number as indicated.Here 11 of a total of 17 genes associated with isolated childhood cancer risk are shown. The remaining 6 are shown in Figure 4 of the main manuscript. Metadata text is faded for all genes where no LoF variants were reported in the included pan-cancer studies. Source data are provided as a Source Data file.
