# Supplementary material for: The evolutionary impact of childhood cancer on the human gene pool
Source: Nat Commun. 2024 Feb 29;15:1881. doi: 10.1038/s41467-024-45975-9 (PMC10904397; doi:10.1038/s41467-024-45975-9)
Supplement: Supplementary file 3 — Description of Additional Supplementary Files [file 41467_2024_45975_MOESM3_ESM.pdf]

## Description of Additional Supplementary Files

**Supplementary Data 1:** A reference sheet providing overview information of each set of Supplementary Data

**Supplementary Data 2:** Combined metadata from included pediatric pancancer studies.

**Supplementary Data 3:** A list of category 1 individual genes and blod category 2 group pediatric cancer predisposition genes with added data as indicated by variable name. Data from source as linked/referenced in Supplementary Data 1 and in the main manuscript.

**Supplementary Data 4:** A list defining genes as linked to pediatric- or adult-onset cancer predisposition syndromes. Based on information as provided in Supplementary Data 3 as linked/referenced in Supplementary Data 1 and in the main manuscript.

**Supplementary Data 5:** Study data Zhang et al. (2015); source as linked/referenced in Supplementary Data 1 and in the main manuscript.

**Supplementary Data 6:** Study data Parsons et al. (2016); source as linked/referenced in Supplementary Data 1 and in the main manuscript.

**Supplementary Data 7:** Study data Mody et al. (2016); source as linked/referenced in Supplementary Data 1 and in the main manuscript.

**Supplementary Data 8:** Study data Oberg et al. (2016); source as linked/referenced in Supplementary Data 1 and in the main manuscript.

**Supplementary Data 9:** Study data Gröbner et al. (2018); source as linked/referenced in Supplementary Data 1 and in the main manuscript.

**Supplementary Data 10:** Study data Wong et al. (2020); source as linked/referenced in Supplementary Data 1 and in the main manuscript.

**Supplementary Data 11:** Study data Byrjalsen et al. (2020); source as linked/referenced in Supplementary Data 1 and in the main manuscript.

**Supplementary Data 12:** Study data Fiala et al. (2021); source as linked/referenced in Supplementary Data 1 and in the main manuscript.

**Supplementary Data 13:** Study data Newmann et al. (2021); source as linked/referenced in Supplementary Data 1 and in the main manuscript.

**Supplementary Data 14:** Study data Stedingk et al. (2021); source as linked/referenced in Supplementary Data 1 and in the main manuscript.

**Supplementary Data 15:** Study data Wagener et al. (2021); source as linked/referenced in Supplementary Data 1 and in the main manuscript.

**Supplementary Data 16:** Study gene panel Zhang et al. (2015); source as linked/referenced in Supplementary Data 1 and in the main manuscript.

**Supplementary Data 17:** Study gene panel Parsons et al. (2016); source as linked/referenced in Supplementary Data 1 and in the main manuscript.

**Supplementary Data 18:** Study gene panel Mody et al. (2016); source as linked/referenced in Supplementary Data 1 and in the main manuscript.

**Supplementary Data 19:** Study gene panel Oberg et al. (2016); source as linked/referenced in Supplementary Data 1 and in the main manuscript.

**Supplementary Data 20:** Study gene panel Gröbner et al. (2018); source as linked/referenced in Supplementary Data 1 and in the main manuscript.

**Supplementary Data 21:** Study gene panel Wong et al. (2020); source as linked/referenced in Supplementary Data 1 and in the main manuscript.

**Supplementary Data 22:** Study gene panel Byrjalsen et al. (2020); source as linked/referenced in Supplementary Data 1 and in the main manuscript.

**Supplementary Data 23:** Study gene panel Fiala et al. (2021); source as linked/referenced in Supplementary Data 1 and in the main manuscript.

**Supplementary Data 24:** Study gene panel Newmann et al. (2021); source as linked/referenced in Supplementary Data 1 and in the main manuscript.

**Supplementary Data 25:** Study gene panel Stedingk et al. (2021); source as linked/referenced in Supplementary Data 1 and in the main manuscript.

**Supplementary Data 26:** Study gene panel Wagener et al. (2021); source as linked/referenced in Supplementary Data 1 and in the main manuscript.

**Supplementary Data 27:** Predicted loss-of-function variants in *ELP1* as reported by gnomad v2.1.1; source as linked/referenced in Supplementary Data 1 and in the main manuscript.

**Supplementary Data 28:** Predicted loss-of-function variants in *ELP1* as reported by Waszak et al. (2020); source as linked/referenced in Supplementary Data 1 and in the main manuscript.

**Supplementary Data 29:** Predicted loss-of-function variants in *GPR161* as reported by gnomad v2.1.1; source as linked/referenced in Supplementary Data 1 and in the main manuscript.

**Supplementary Data 30:** Predicted loss-of-function variants in *GPR161* as reported by Begemann et al. (2020); source as linked/referenced in Supplementary Data 1 and in the main manuscript.

**Supplementary Data 31:** Variant information from National Center for Biotechnology Information. ClinVar. Search terms: *SAMD9* [gene] "single nucleotide" & "Pathogenic"; source as linked/referenced in Supplementary Data 1 and in the main manuscript.

**Supplementary Data 32:** Variant information from National Center for Biotechnology Information. ClinVar. Search terms: *SAMD9L* [gene] "single nucleotide" & "Pathogenic"; source as linked/referenced in Supplementary Data 1 and in the main manuscript.

**Supplementary Data 33:** Variant information from National Center for Biotechnology Information. ClinVar. Search terms: HRAS [gene] "single nucleotide" & "Pathogenic"; source as linked/referenced in Supplementary Data 1 and in the main manuscript.

**Supplementary Data 34:** Predicted loss-of-function variants in *MSH2* as reported by gnomad v2.1.1; source as linked/referenced in Supplementary Data 1 and in the main manuscript.

**Supplementary Data 35:** Predicted loss-of-function variants in *DIS3L2* as reported by gnomad v2.1.1; source as linked/referenced in Supplementary Data 1 and in the main manuscript.

**Supplementary Data 36:** Metadata on GWAS findings related to cancer susceptibility in humans; source as linked/referenced in Supplementary Data 1 and in the main manuscript.
