# Supplementary material for: The evolutionary impact of childhood cancer on the human gene pool
Source: Nat Commun. 2024 Feb 29;15:1881. doi: 10.1038/s41467-024-45975-9 (PMC10904397; doi:10.1038/s41467-024-45975-9)
Supplement: Supplementary file 5 — Reporting Summary [file 41467_2024_45975_MOESM5_ESM.pdf]

nature portfolio

Corresponding author(s): Ulrik Kristoffer Stoltze

Last updated by author(s): Jun 29, 2023

Reporting Summary

Nature Portfolio wishes to improve the reproducibility of the work that we publish. This form provides structure for consistency and transparency in reporting. For further information on Nature Portfolio policies, see our Editorial Policies and the Editorial Policy Checklist.

Statistics

For all statistical analyses, confirm that the following items are present in the figure legend, table legend, main text, or Methods section.

n/a	Confirmed	
☐	☒	The exact sample size (*n*) for each experimental group/condition, given as a discrete number and unit of measurement
☐	☒	A statement on whether measurements were taken from distinct samples or whether the same sample was measured repeatedly
☐	☒	The statistical test(s) used AND whether they are one- or two-sided<br>*Only common tests should be described solely by name; describe more complex techniques in the Methods section.*
☐	☒	A description of all covariates tested
☐	☒	A description of any assumptions or corrections, such as tests of normality and adjustment for multiple comparisons
☐	☒	A full description of the statistical parameters including central tendency (e.g. means) or other basic estimates (e.g. regression coefficient) AND variation (e.g. standard deviation) or associated estimates of uncertainty (e.g. confidence intervals)
☐	☒	For null hypothesis testing, the test statistic (e.g. *F*, *t*, *r*) with confidence intervals, effect sizes, degrees of freedom and *P* value noted<br>*Give P values as exact values whenever suitable.*
☒	☐	For Bayesian analysis, information on the choice of priors and Markov chain Monte Carlo settings
☒	☐	For hierarchical and complex designs, identification of the appropriate level for tests and full reporting of outcomes
☒	☐	Estimates of effect sizes (e.g. Cohen's *d*, Pearson's *r*), indicating how they were calculated

Our web collection on statistics for biologists contains articles on many of the points above.

Software and code

Policy information about availability of computer code

Data collection	No original data collection was performed for this study.
Data analysis	R version 4.0.2 (2020-06-22) & RStudio (version 2022.07.1). The full, commented code used for data wrangling, analysis, and visualization is available at (https://github.com/TruthSeqer/peds_cancer_genes_vs_constraint/blob/main/constraint_in_pCPS_genes_revised.Rmd). The scripts are preambled by the required packages, which, by name(version number), are; readxl(1.4.1), backports(1.4.1), systemfonts(1.0.4), plyr(1.8.8), lazyeval(0.2.2), splines(4.2.2), TH.data(1.1-1), digest(0.6.31), htmltools(0.5.4), fansi(1.0.3), memoise(2.0.1), googlesheets4(1.0.1), tzdb(0.3.0), penxlsx(4.2.5.1), remotes(2.4.2), modelr(0.1.10), matrixStats(0.63.0), sandwich(3.0-2), prettyunits(1.1.1), colorspace(2.0-3), vest(1.0.3), textshaping(0.3.6), haven(2.5.1), xfun(0.36), callr(3.7.3), crayon(1.5.2), jsonlite(1.8.4), libcoin(1.0-9), Exact(3.2,) survival(3.4-0), glue(1.6.2), polyclip(1.10-4), gtable(0.3.1), gargle(1.2.1), pkgbuild(1.4.0), clipr(0.8.0), Quandl(2.11.0), mvtnorm(1.1-3), DBI(1.1.3), miniUI(0.1.1).1, Rcpp(1.0.9), xtable(1.8-4), gridtext(0.1.5), foreign(0.8-83), bit(4.0.5), proxy(0.4-27), stats4(4.2.2), profvis(0.3.7), htmlwidgets(1.6.0), httr(1.4.4), modeltools(0.2-23), ellipsis(0.3.2), farver(2.1.1), urlchecker(1.0.1), pkgconfig(2.0.3), ultcompView(0.1-8), dbplyr(2.2.1), utf8(1.2.2), labeling(0.4.2), tidyselect(1.2.0), rlang(1.0.6), later(1.3.0), munsell(0.5.0), cellranger(1.1.0), tools(4.2.2), cachem(1.0.6), generics(0.1.3), broom(1.0.2), evaluate(0.19,) fastmap(1.1.0), ragg(1.2.4), yaml(2.3.6), processx(3.8.0), bit64(4.0.5), fs(1.5.2), zip(2.2.2), coin(1.4-2), rootSolve(1.8.2).3, mime(0.12,) xml2(1.3.3), compiler(4.2.2), rstudioapi(0.14,) curl(4.3.3), e1071(1.7-12), gt(0.8.0), reprex(2.0.2), tweenr(2.0.2), broom.helpers(1.11.0), DescTools(0.99.47), stringi(1.7.8), ps(1.7.2), lattice(0.20-45), Matrix(1.5-1), vctrs(0.5.1), pillar(1.8.1), lifecycle(1.0.3), lmtest(0.9-40), ata.table(1.14.6), lmom(2.9,) httpuv(1.6.7), R6(2.5.1), promises(1.2.0).1, gld(2.6.6), sessioninfo(1.2.2), codetools(0.2-18), boot(1.3-28), MASS(7.3-58.1), assertthat(0.2.1), pkgload(1.3.2), withr(2.5.0), nortest(1.0-4), multcomp(1.4-20), expm(0.999-6), parallel(4.2.2), hms(1.1.2), quadprog(1.5-8), class(7.3-20), rmarkdown(2.19,) googledrive(2.0.0), shiny(1.7.4).

For manuscripts utilizing custom algorithms or software that are central to the research but not yet described in published literature, software must be made available to editors and reviewers. We strongly encourage code deposition in a community repository (e.g. GitHub). See the Nature Portfolio guidelines for submitting code & software for further information.

Data

Policy information about availability of data

All manuscripts must include a data availability statement. This statement should provide the following information, where applicable:

- Accession codes, unique identifiers, or web links for publicly available datasets
- A description of any restrictions on data availability
- For clinical datasets or third party data, please ensure that the statement adheres to our policy

The raw data used in this study are fully available without any restrictions, either in the supplementary datasets or via the links provided both below and in the study code (see Code availability). Generated in this study are provided in the Supplementary Information/Source Data file. Exceptions are GnomAD v2.1 exome data ("All chromosomes sites VCF", https://storage.googleapis.com/gcp-public-data--gnomad/release/2.1.1/vcf/exomes/gnomad.exomes.r2.1.1.sites.vcf.bgz); associated coverage data available from https://storage.googleapis.com/gcp-public-data--gnomad/release/2.1/coverage/exomes/gnomad.exomes.coverage.summary.tsv.bgz; associated downsampling data from https://storage.googleapis.com/gcp-public-data--gnomad/release/2.1.1/constraint/gnomad.v2.1.1.lof_metrics.downsamplings.txt.bgz; constraint metrics from Supplementary Dataset 11 from Karczewski et al. (Nature, 2020, https://static-content.springer.com/esm/art%3A10.1038%2Fs41586-020-2308-7/MediaObjects/41586_2020_2308_MOESM4_ESM.zip); cancer predisposition syndrome gene list - available in the suppl. for Rahman (Nature, 2014, https://www.ncbi.nlm.nih.gov/pmc/articles/PMC4975511/); and CPS mutations in adults from suppl. data 2 from Huang et al. (Cell, 2018, https://www.ncbi.nlm.nih.gov/pmc/articles/PMC5949147/). These links are also provided, when needed, in the comments for the code (see Code availability).

Research involving human participants, their data, or biological material

Policy information about studies with human participants or human data. See also policy information about sex, gender (identity/presentation), and sexual orientation and race, ethnicity and racism.

Reporting on sex and gender	Analysis did not include sex or gender variables.
Reporting on race, ethnicity, or other socially relevant groupings	Analysis did not include race, ethnicity, or other socially relevant grouping variables.
Population characteristics	This study did not include original data. For metrics on population characteristics, we defer to the original studies.
Recruitment	This study did not recruit participants.
Ethics oversight	This study did not include original data and, as such, did not qualify for nor require ethical oversight.

Note that full information on the approval of the study protocol must also be provided in the manuscript.

Field-specific reporting

Please select the one below that is the best fit for your research. If you are not sure, read the appropriate sections before making your selection.

☒ Life sciences ☐ Behavioural & social sciences ☐ Ecological, evolutionary & environmental sciences

For a reference copy of the document with all sections, see nature.com/documents/nr-reporting-summary-flat.pdf

Life sciences study design

All studies must disclose on these points even when the disclosure is negative.

Sample size	Sample sizes was determined by 1) the pan-cancer studies that fulfilled the inclusion criteria and 2) single nucleotide variant metadata from 141,456 adult humans aggregated by gnomAD; including their calculations of mutational constraint. This provided sufficient data to support the reported results and conclusion, given the limitations presented and discussed.
Data exclusions	No relevant data were excluded.
Replication	Reproducability is ensured through full availability of both the code and required data.
Randomization	Not applicable. This is a metaanalysis of observational germline genetics.
Blinding	Not applicable. This is a metaanalysis of observational germline genetics.

Reporting for specific materials, systems and methods

We require information from authors about some types of materials, experimental systems and methods used in many studies. Here, indicate whether each material, system or method listed is relevant to your study. If you are not sure if a list item applies to your research, read the appropriate section before selecting a response.

Materials & experimental systems

n/a	Involved in the study
☒	☐ Antibodies
☒	☐ Eukaryotic cell lines
☒	☐ Palaeontology and archaeology
☒	☐ Animals and other organisms
☒	☐ Clinical data
☒	☐ Dual use research of concern
☒	☐ Plants

Methods

n/a	Involved in the study
☒	☐ ChIP-seq
☒	☐ Flow cytometry
☒	☐ MRI-based neuroimaging
